# Noncontact Damage Topography Reconstruction by Wavenumber Domain Analysis Based on Air-Coupled Ultrasound and Full-Field Laser Vibrometer

**DOI:** 10.3390/s21020609

**Published:** 2021-01-17

**Authors:** Hui Zhang, Dongmei Liang, Xiaobo Rui, Zhuochen Wang

**Affiliations:** State Key Laboratory of Precision Measurement Technology and Instrument, Tianjin University, Tianjin 300072, China; hzhang@tju.edu.cn (H.Z.); dm_liang@tju.edu.cn (D.L.); zc_wang@tju.edu.cn (Z.W.)

**Keywords:** damage evaluation, air-coupled ultrasound, Lamb wave, laser vibrometer, wavenumber domain analysis

## Abstract

Noncontact ultrasonic detection technology is an effective method to detect damage in time. This paper proposes a noncontact damage detection system based on air-coupled ultrasound and full-field laser vibrometer, which realizes the excitation of relatively single-mode guided waves and the wavefield automatic detection. The system performance is verified through experiments, and the experimental wavenumber is consistent with the theoretical dispersion characteristics of the Lamb wave in the A0 mode. Based on this system, the topography reconstruction algorithms, including the Wavenumber Filtering Algorithm and Spatial Wavenumber Algorithm, were tested and compared with the aluminum alloy plate and the carbon fiber reinforced polymer plate. The results show that, based on the air-coupled ultrasound and full-field laser vibrometer detection system, the Spatial Wavenumber Algorithm has better imaging error and contrast, and the damage edge detection is smoother.

## 1. Introduction

Ultrasonic technology is widely used in structural damage assessment due to its advantages of nondestructive and fast testing [1,2,3]. It can evaluate the health status of the structure during the whole life cycle of production, use and maintenance. Ultrasound has a wide frequency range and various waveforms, which provide abundant possibilities for nondestructive testing [4,5]. In recent years, noncontact ultrasonic nondestructive testing technology has received widespread attention, because of its advantages of no coupling, no surface contact, and fast scanning [6,7].

Electro-Magnetic Acoustic Transducer (EMAT) technology, laser ultrasound and air-coupled ultrasound are currently three widely used noncontact ultrasonic detection methods. EMAT technology is suitable for thickness measurement and defect characterization of various metal pipes or plates, and can be applied under high-temperature conditions [8]. The low energy conversion efficiency, the short distance between the coil and the tested structure limit the application and development of related technologies, and the material to be tested is required to have good conductivity or ferromagnetism [9].

Laser ultrasound technology uses pulsed lasers to achieve high-frequency ultrasonic excitation, based on thermo-elastic and thermo-erosion effects. However, the conversion efficiency of photoacoustic energy is not high, and the surface of the material will be damaged when the laser energy is too high. In laser ultrasound, laser Doppler vibrometers are often used as testing equipment, and the Doppler frequency shift phenomenon is used to measure the in-plane or out-of-plane surface vibration displacement and velocity [10]. The laser-related technology can perform spatial distributed detection, has a high spatial resolution, and can detect large-size or complex curved surfaces [11,12].

Air-coupled ultrasound technology uses air as the coupling medium between the transducer and the structure, which can generate bulk waves, surface waves and also Lamb waves. Among them, the Lamb waves in a thin-plate structure can be propagated for a long distance with low attenuation energy [13]. Air-coupled Lamb wave detection methods have been extensively studied, including visualization of impact, delamination, debonding, and surface damage imaging of metal and composite plates. The main limitation of air-coupled ultrasonic transducers is the enormous acoustic impedance mismatch between the piezoelectric material and air. Researchers are also coming up with new ways to overcome the problem [14,15]. Each detection technology has its unique advantages and limitations. Hybrid detection systems have the potential to combine multiple advantages and to break through the bottleneck of existing technologies, such as laser ultrasound combined with electromagnetic ultrasound, piezoelectric ultrasound and laser ultrasound, etc., [16].

For structural damage detection, the damage topography reconstruction of plate-shaped structures is the most concerned. Typical algorithms include reconstruction algorithm of probabilistic inspection (RAPI) [17], single-transmitter and multireceiver method (STMR) [18], wavenumber domain imaging [19], etc. Among them, RAPI and STMR are mainly based on the difference in the energy passability of damage to achieve location and topography reconstruction. The wavenumber domain imaging method analyzes the full wavefield data of the range to be measured by sampling in the time–space domain, and the signal information is more abundant and accurate [20,21].

Juarez et al. came up with a methodology based on multifrequency local wavenumber analysis for quantitative assessment of multi-ply delamination damage, including the delamination size and ply depth in carbon fiber reinforced polymer (CFRP) composite specimens [22]. Tian et al. presented a Spatial Wavenumber Algorithm through global inspection by phased array beamforming and local damage evaluation via wavenumber analysis [23]. The detected delamination location, size and shape agree well with those of an ultrasonic C-scan, and the total inspection time was reduced greatly. Jeon et al. implemented two signal processing methods, including Local Wavenumber Mapping (LWM) and Acoustic Wavenumber Spectroscopy (AWS), to determine dominant wavenumber components of the measured wavefield [24]. In this way, the structural delamination and debonding damage were clearly and accurately identified. Kudela et al. aimed at enhancing damage visualization in thin-walled structures, through a combined system of the PZT transducer and the laser Doppler vibrometer [25]. They proposed a Wavenumber Filtering Algorithm for the estimation of the length and orientation of the crack. After that, Kudela et al. used wavenumber adaptive image filtering and signal processing to visualize and assess the impact-induced damage location and size [26]. The results showed that damage caused by the impact of 10 J or higher could be successfully detected using the presented approach. Radzienski et al. developed a detection technique, where guided waves were excited by PZT transducer, and measured by scanning laser vibrometer [27]. By means of mapping irregularities of propagating guided waves, damage characteristics in various types of composite plates were obtained. The above-mentioned methods usually use the traditional contact PZT combined with a vibrometer to complete the analysis in the wavenumber domain, which lacks the flexibility of detection. So a hybrid technology using air-coupled transmitters and laser receivers was proposed. Harb et al. confirmed that using this system and implementing the simple Snell’s law method is highly sensitive and effective in characterizing the dispersion curves of Lamb waves in composite structures as well as its angular dependency [28]. In addition, they illustrated the feasibility of using the hybrid system and the guided wave zero-lag cross-correlation imaging condition in characterizing the barely visible impact damages in thin composite structures [29].

This paper uses a noncontact combined detection scheme, including air-coupled ultrasound and laser vibrometer. The air-coupled ultrasonic technology can flexibly excite the Lamb wave of a specific mode to achieve long-distance and high-sensitivity detection. Laser vibrometer technology can obtain the vibration of the structure surface and realize the full wavefield data detection with high spatial resolution. The above system is applied to the damage topography reconstruction for metal plates and composite material plates for testing. The Spatial Wavenumber Algorithm and the Wavenumber Filtering Algorithm are compared by the noncontact system. The system has the characteristics of flexible operation and fast scanning, and can be combined with a variety of algorithms, and has the application potential for rapid scanning of large-scale equipment structures.

## 2. Air-Coupled Ultrasonic and Full-Field Laser Vibrometer Detection System

### 2.1. System Composition and Function

**Figure 1:**
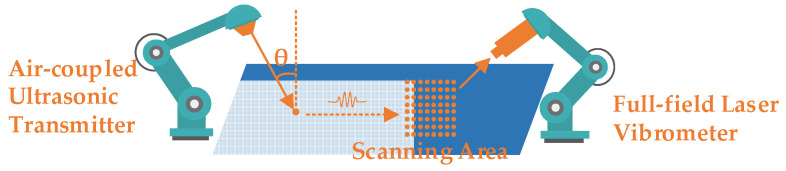
The air-coupled ultrasonic and full-field laser vibrometer detection system diagram.

The noncontact detection system proposed in this paper is shown in Figure 1, which mainly includes an air-coupled ultrasonic transmitter and a full-field laser vibrometer. The air-coupled ultrasonic transmitter is responsible for exciting the different modal ultrasonic waves by adjusting the incident angle. Laser vibration measurement technology is responsible for realizing the distributed point scanning measurement with high spatial resolution. The two are applied to the excitation and the receiving parts, which can give full play to their advantages to conduct the guided wave excitation and the full-wave field data acquisition. The sheet structural damage can be characterized by the wave field changes by different algorithms.

**Figure 2:**
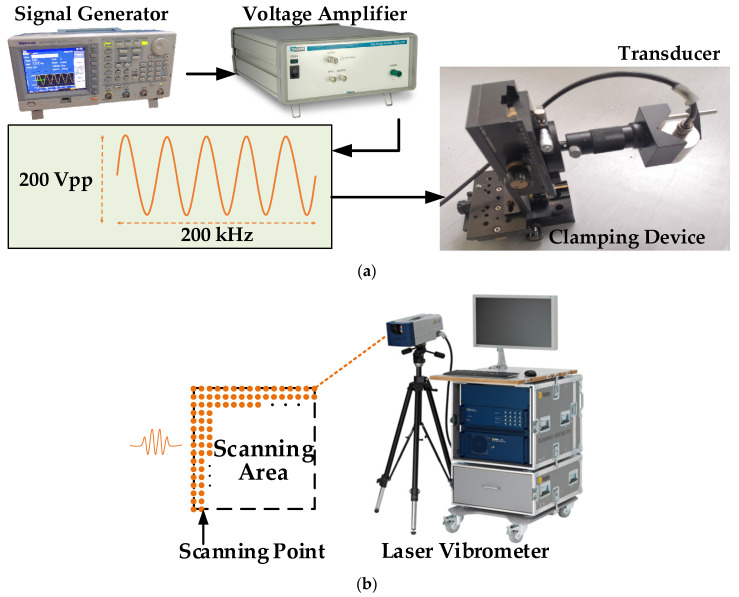
The specific composition of the system in the experiment: (**a**) the ultrasonic transmitting component; (**b**) The signal receiving component.

Specifically, in the subsequent experimental system, the ultrasonic transmitting component is shown in Figure 2a, including a signal generator (Tektronix AFG3152C), a voltage amplifier (Tegam Model 2340) and an air-coupled ultrasonic transducer (Ultran Group NCG200-D25). The transducer is a circular noncontact transducer with a center frequency of 200 kHz and a bandwidth of 40% of the center frequency. It is fixed by a clamping device and emits ultrasonic waves to the surface of the thin plate. The incident angle of the transducer is easy to accurately adjust. According to Snell’s law of refraction, the ultrasonic Lamb wave with a relatively single mode is excited by a specific angle. The out-of-plane displacement of the A0 mode wave is more obvious than the in-plane displacement and is easy to actuate, thus the A0 mode Lamb wave is usually considered as the preferred mode for damage detection. Because of the dispersion characteristics of the Lamb wave in the plate, the higher the frequency is, the more complex the lamb wave modes will be. In order to reduce the number of wave modes as much as possible to obtain the relatively pure guided wave, a lower frequency of 200 kHz is selected as the excitation frequency of the probe. The signal generator outputs a 1 ms, 4 Vpp, 5 cycles, 200 kHz sine pulse train, and 50 times amplified to realize the air-coupled ultrasonic excitation.

The signal receiving component of the system is shown in Figure 2b, which is performed by a full-field laser Doppler vibrometer (PSV-500-V). The system consists of one laser head, a data acquisition system with velocity and displacement decoders as well as a signal generator, and a data management system. The He-Ne laser of the system has a wavelength of 633 nm, the velocity resolution is 0.01 μm/s (1 Hz), a minimum displacement resolution can be up to pico-meter level, and the frequency measurements range from DC to 25 MHz. The Doppler frequency shift effect is used to realize the measurement of the surface velocity or displacement, with high spatial resolution, scanning speed, and measurement accuracy. The excitation position of the air-coupled sensor is fixed, and the laser vibrometer is synchronously triggered to receive the response of a point every excitation time. In this way, the Lamb wave field of the area to be measured is collected point by point, so as to detect the Lamb wave propagation at any point at any time. This laser scanning measurement method can replace the method with a large number of sensors, greatly reducing the complexity of the experiment operation and improving the spatial resolution of the signal. In the experiment, the time-domain measurement mode is used, the sampling frequency is 1.25 MHz, the range of the band-pass filter is 100~300 kHz, and the trigger acquisition signal is an external input analog signal from the signal generator. In order to ensure the high precision of laser vibration measurement and improve the signal-to-noise ratio, each point is measured 32 times and the average is taken as the result.

### 2.2. Excitation and Receiving Performance Testing of the System

#### 2.2.1. Aluminum Alloy Plate

An aluminum alloy plate with a density of 2700 kg/m^3^, Young’s modulus of 71.7 GPa, a Poisson’s ratio of 0.33, and a thickness of 2 mm is used for the system test. Lamb waves in thin plates have dispersion phenomena, that is, the propagation speed of sound waves along the surface is a function of frequency or wavelength. The phase velocities and group velocities of different wave modes at different frequencies can be obtained according to the Rayleigh-Lamb wave equation. It can be determined that the phase velocity of the A0 mode Lamb wave at a frequency of 200 kHz is 1744 m/s, thereby determining that the optimal incident angle of the A0 mode Lamb wave excitation is about 11°14′.

**Figure 3:**
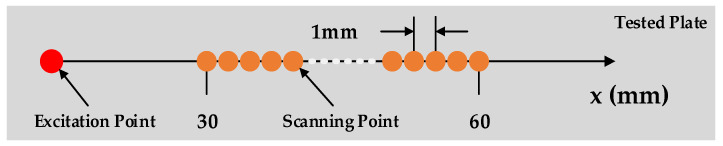
Schematic diagram of linear scanning of laser vibrometer.

The driving ability of a pure fundamental A0 mode at the selected incident angle by the air-coupled ultrasonic transducer, and the ability to acquire the wavefield in the plate by the full-field laser vibrometer are tested. The distance between the air-coupled transducer and the plate surface is about 40 mm. The wavefield u(t, x) of the Lamb wave in the plate is obtained by linear scanning of the laser Doppler vibrometer. The scanning range is about 30 mm~60 mm from the excitation point, with an interval of 1 mm, as shown in Figure 3.

**Figure 4:**
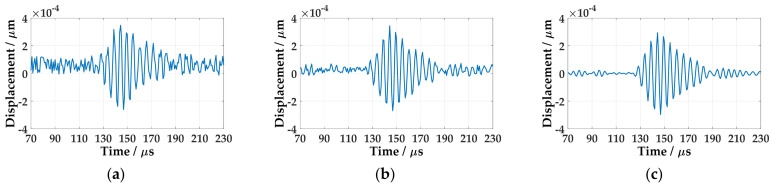
The wave at 40 mm in aluminum plate: (**a**) Single acquisition signal; (**b**) Average of 32 acquisitions; (**c**) After filtering.

The time-domain waveform data from the 40 mm are selected to analyze, as shown in Figure 4. It can be seen that the signal noise in one acquisition is large, as in Figure 4a. The random noise is reduced after averaging 32 times of data, as in Figure 4b. After band-pass filtering, the noise is further reduced, as in Figure 4c.

**Figure 5:**
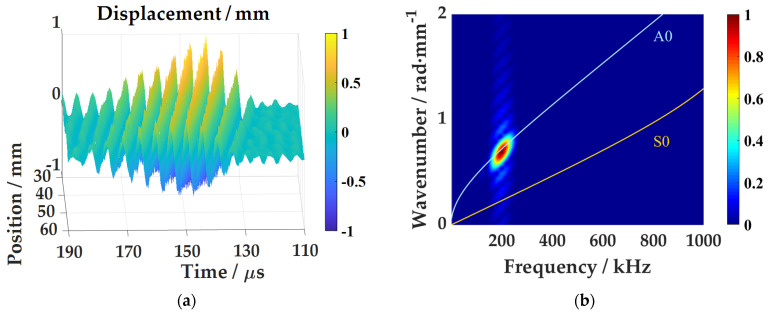
Test results of 2 mm thick aluminum plate: (**a**) Lamb wave one-dimensional time domain wave field; (**b**) Frequency-wavenumber spectrum.

Based on the above data, the wavefield at different moments of the one-dimensional straight line in the aluminum plate can be obtained, as shown in Figure 5a. The air-coupled Lamb wave spreads out in the range of about 50 μs in the time domain. The wave moves forward, and the propagation speed of the wave is the same during the whole process. The time-domain wavefield data are transformed into the frequency-wavenumber domain by two-dimensional Fourier transform for characterization, and compared with the theoretical spectrum, as shown in Figure 5b. It can be seen that the center frequency of the received lamb wave is about 200 kHz, and the center wavenumber is about 0.72 rad/mm. Almost only the A0 mode Lamb wave is excited, which is consistent with the target. The above experiments confirmed that the system can excite a relatively pure A0 mode Lamb wave in the aluminum plate by exciting at the theoretically optimal angle. However, the ability to acquire the wavefield data has also been verified.

#### 2.2.2. Carbon Fiber Reinforced Polymer(CFRP) Plate

**Figure 6:**
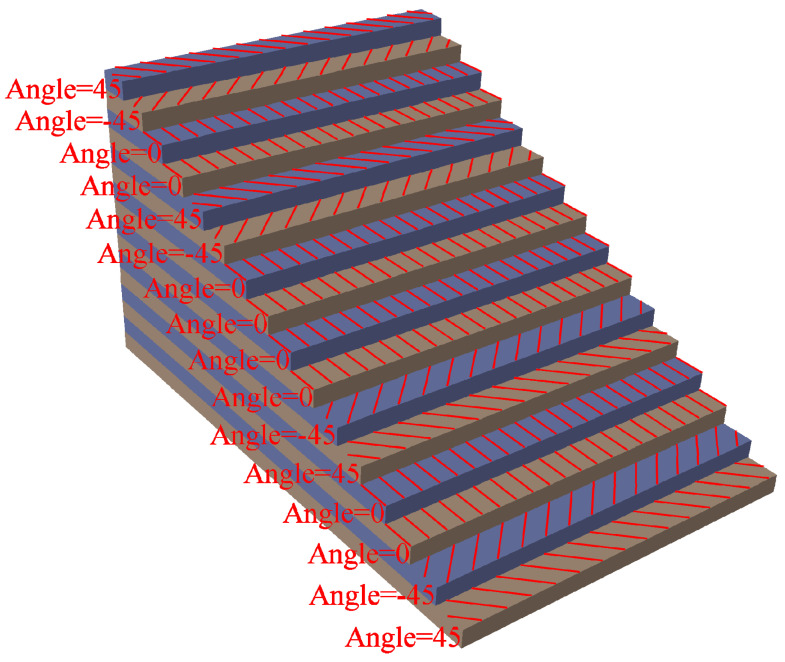
Schematic diagram of CFRP plate laying scheme.

For the CFRP plate, the characteristic parameters are shown in Table 1, which is a 16-layer composite plate layered in [+45/−45/0/0]_2s_, as shown in Figure 6.

**Figure 7:**
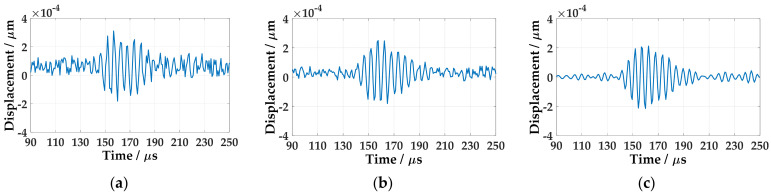
The wave at 40 mm in CFRP plate: (**a**) Single acquisition signal; (**b**) Average of 32 acquisitions; (**c**) After filtering.

For multilayer composite materials, due to its heterogeneity, inherent anisotropy of the material and multilayer structure, the propagation of waves in composite materials is very complicated. In this paper, the dispersion relationship between the symmetric wave mode and the antisymmetric wave mode in the thin plate is obtained from the three-dimensional elastic theory, and it is extended to composite laminates with arbitrary stacking order. The phase velocity of the A0 mode Lamb wave at 200 kHz frequency is 1598 m/s, and the optimal incident angle of the A0 mode Lamb wave at this frequency is about 12°17′. The experiment process is the same as that of the aluminum plate, and the results are as shown in Figure 7.

**Figure 8:**
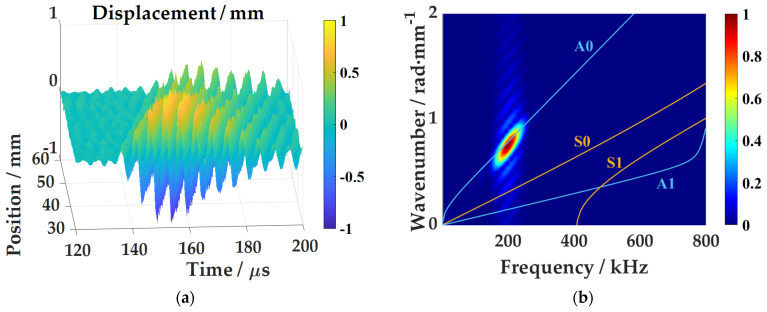
Test results of 2 mm thick CFRP plate: (**a**) Lamb wave one-dimensional time domain wave field; (**b**) Frequency-wavenumber spectrum.

The one-dimensional linear time-domain wavefield and frequency-wavenumber spectrum in the CFRP plate are shown in Figure 8. The Lamb wave propagates straight forward and the slope remains the same, indicating that the wave propagation speed is the same throughout the process. According to the comparison between the theoretical frequency-wavenumber spectrum and the experimental wavenumber spectrum, it is found that the center frequency of this mode Lamb wave is about 200 kHz, and the center wavenumber is about 0.78 rad/mm. The experiments have proved that through excitation at the theoretical optimal angle of incidence, a relatively pure A0 mode Lamb wave can also be obtained in the composite plate.

## 3. Topography Reconstruction Algorithms

### 3.1. Wavenumber Filtering Algorithm (WFA)

WFA processes the full wavefield information through filtering in the wavenumber domain, and then the filtered time domain wavefield is obtained through the inverse Fourier transform, and the damage situation is reflected by the time domain energy signal [20].

(1) Perform a two-dimensional Fourier transform on the acquired wavefield data *v*(*x*,*y*,*t*) of each point, and transform it from the spatial domain (*x*,*y*) to the wavenumber domain (*k_x_*,*k_y_*):

(1) $$V(k_{x},k_{y},t)=\int_{-\infty }^{+\infty }\int_{-\infty }^{+\infty }v(x,y,t)e^{-j(k_{x}x+k_{y}y)}dxdy,$$

where *v*(*x*,*y*,*t*) is the out-of-plane velocity, and *V*(*k_x_*,*k_y_*,*t*) is the wavefield data in wavenumber domain.

(2) The wavenumber domain pattern of propagating guided waves is defined in the wavenumber domain according to the selected number of wavefield images. Calculate the average wavenumber spectrum *V_avg_*(*k_x_*,*k_y_*) of the wavefield data, which represents the propagation of waves in this period of time in the wavenumber domain, which can be defined as:

(2) $$V_{avg}(k_{x},k_{y})=\frac{1}{M}\sum_{t}V(k_{x},k_{y},t),$$

where *M* is the total number of time points of wavefield data.

(3) According to the distribution of the average wavenumber spectrum *V_avg_*(*k_x_*,*k_y_*), the threshold filter function *M*(*k_x_*,*k_y_*) is calculated as follows:

(3) $$M(k_{x},k_{y})=\left\{ \begin{array}{l} 0\begin{array}{cc} & V_{avg}(k_{x},k_{y})>threshold \end{array}\\ 1\begin{array}{cc} & \mathrm{otherwise} \end{array} \end{array}\right.,$$

The purpose of the filter function is to remove the mainstream health signals in the wavefield and retain the unhealthy signals caused by damage. Since the wavenumber intensity of the mainstream health signal in the wavenumber domain is larger, and the wavenumber intensity of the damage signal is smaller, the wavenumber with the larger intensity value is filtered out, and the smaller one is retained, thereby completing the threshold filtering. The optimal value of the threshold in the function is not known a priori. In the experiment, according to the cumulative distribution of the average wavenumber spectrum *V_avg_*(*k_x_*,*k_y_*), the wavenumber intensity value with a larger amplitude is selected as the candidate threshold. While ensuring the damage detection effect, reduce noise interference as much as possible. Select the candidate threshold that can make 5% of the element values in the filter function matrix 0 as the final experimental threshold to realize the automatic setting of the threshold.

(4) The generated threshold filter function is multiplied by the wavefield image at each moment in the wavenumber domain to achieve filtering. It is important to note that the same filter function is applied to each wavefield image:

(4) $$\bar{V}(k_{x},k_{y},t)=V(k_{x},k_{y},t)M(k_{x},k_{y}),$$

(5) Subsequently, the wavefield data filtered by each threshold are subjected to a two-dimensional inverse Fourier transform to obtain a wavefield image in the time–space domain after filtering:

(5) $$\bar{v}(x,y,t)=F_{2D}^{-1}\{\bar{V}(k_{x},k_{y},t)\},$$

(6) The proposed algorithm given by Equations (1)–(5) eliminates the main components of guided wave, mainly including incident wave. All structural features and damage locations may be easily identified. Repeat the above process for all wavefield data and merge all filtered data with root mean square (RMS) as:

(6) $$RMS_{x,y}=\sqrt{\frac{1}{N}\sum_{t=1}^{N}\bar{v}{\left(x,y,t\right)}^{2}},$$

Through the filtered Root Mean Square energy field, the structural features and damage locations can be easily identified.

### 3.2. Spatial Wavenumber Algorithm (SWA)

SWA directly reflects the damage situation through the change of wavenumber [18]. The three-dimensional Fourier Transform is performed on the time-domain full-field data combining the spatial window function to obtain spatial wavenumber information at different frequencies. The specific frequency range is selected according to the excitation signal bandwidth, and average the wavenumber signals in the entire frequency band. The average spatial wavenumber diagram is used to identify the damage. This method synthesizes the changes of the wavenumber at each frequency and effectively contains most of the damage information.

(1) Perform a short-space three-dimensional Fourier transform on the wavefield data *v*(*x*,*y*,*t*) at different spatial positions to obtain the frequency-wavenumber spectrum. The short-space three-dimensional Fourier transform is a direct extension of short-time Fourier transform to multidimensional problems, that is, the time–space wave field is decomposed into small segments in two-dimensional space before the Fourier transform. To do this, the wavefield data are multiplied by a window function that is nonzero only for a short period in space, but is constant throughout the time dimension. Then three-dimensional Fourier transform is applied to the generated wavefield segments. The specific transform form is as follows:

(7) $$S(x_{0},y_{0},k_{x},k_{y},f)=\int_{-\infty }^{\infty }\int_{-\infty }^{\infty }v(x,y,t)W(x-x_{0},y-y_{0})e^{-j(2\pi ft+k_{x}x+k_{y}y)}dtdxdy,$$

where (*x*_0_,*y*_0_) is the reserved spatial position point, and *W*(*x-x*_0_,*y-y*_0_) is the spatial window function based on the two-dimensional Hanning window:

(8) $$W(x,y)=\left\{ \begin{array}{l} 0.5\times [1+\cos(\frac{2\pi \sqrt{x^{2}+y^{2}}}{D})]\begin{array}{cc} & \sqrt{x^{2}+y^{2}}\leq \frac{D}{2} \end{array}\\ 0\begin{array}{cc} \begin{array}{ccc} & & \;\;\;\;\;\;\;\;\;\sqrt{x^{2}+y^{2}}>\frac{D}{2} \end{array} & \end{array} \end{array}\right.,$$

where *D* is the length of the spatial window. By sliding the window function along the spatial dimension, the frequency-wavenumber spectrum can be obtained.

(2) According to the space-frequency-wavenumber spectrum obtained, find the maximum wavenumber vector of each spatial location point under each frequency component:

(9) $$k^{*}(x_{0},y_{0},f)=\arg\underset{k}{\max}\left|S(x_{0},y_{0},k_{x},k_{y},f)\right|,$$

(3) According to the frequency band range of the excitation signal, the average spatial maximum wavenumber value is calculated as:

(10) $$\bar{k^{*}}(x_{0},y_{0})=\frac{1}{N}\sum_{i=1}^{N}\left|k^{*}(x_{0},y_{0},f_{i})\right|,$$

where *f*_i_ is a frequency during the selected range. Hence the average spatial wavenumber value represents the wavenumber at each location. The wavenumber diagram of the entire space is obtained, and displaying the damage imaging situation through the direct change of the wavenumber.

## 4. Results and Discussion

### 4.1. Experiment Platform

**Figure 9:**
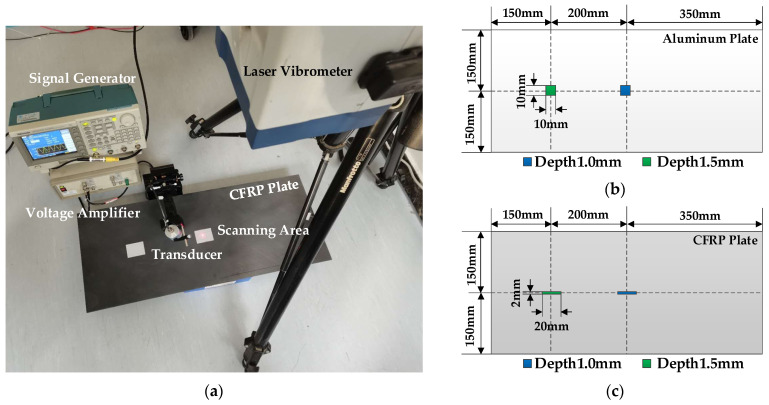
Experiment platform: (**a**) Experimental setup for the detection and estimation of damage on the CFRP plate; (**b**) The damage information of the tested aluminum plate; (**c**) The damage information of the tested CFRP plate.

The experimental site and the damage information of the tested plates are shown in Figure 9.

The aluminum plate and CFRP plate are tested in the experiment. The damages are processed on the surface of plates by cutting grooves. The cutting depth is 1 mm and 1.5 mm respectively. The preset damage size of aluminum plate is 10 mm × 10 mm × 1 mm and 10 mm × 10 mm × 1.5 mm, and the preset damage size of composite plate is 20 mm × 2 mm × 1 mm and 20 mm × 2 mm × 1.5 mm. The scanning range of the aluminum plates is 40 mm × 40 mm, and the scanning range of the composite plates is 60 mm × 20 mm. The scanning center point is coincident with the damage center point, the sampling point interval is 1 mm, and the sampling frequency is 1.25 MHz, the total sampling time for one scanning point is 500 μs.

### 4.2. Topography Reconstruction Process

Taking the CFRP plate with 20 mm × 2 mm × 1 mm damage as an example, the data processing processes of the WFA and SWA are shown below.

(1) WFA

**Figure 10:**
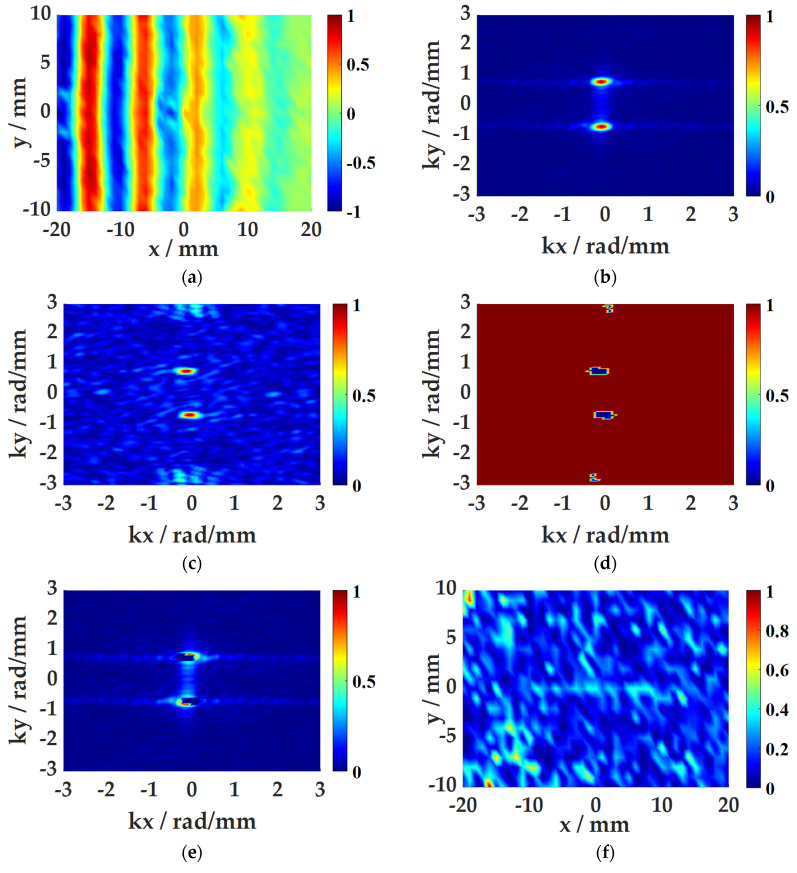
WFA process: (**a**) space-time domain data; (**b**) wavenumber domain spectrum; (**c**) average wavenumber spectrum; (**d**) the threshold filter function; (**e**) wavenumber domain data after filtering (**f**) time–space domain data after filtering.

The time–space three-dimensional wavefield data are subjected to a two-dimensional Fourier transform to obtain the wavenumber distribution diagram at different times. Figure 10a,b are the time–space domain and wavenumber domain data at *t* = 136 μs. Sum and average all wavenumber domain data to obtain the average wavenumber spectrum as shown in Figure 10c. Based on the average wavenumber spectrum data, select the threshold that can make 5% of the data in the filter function zero, and get the threshold filter function as Figure 10d. Finally, the threshold filter function is applied to the wavenumber data at each time point, and a two-dimensional inverse Fourier transform is performed. Figure 10e,f are the wavenumber data and the time–space data after filtering at *t* = 136 μs. The filtered space-time domain data are combined with the root mean square energy function to obtain the final damage imaging.

(2) SWA

**Figure 11:**
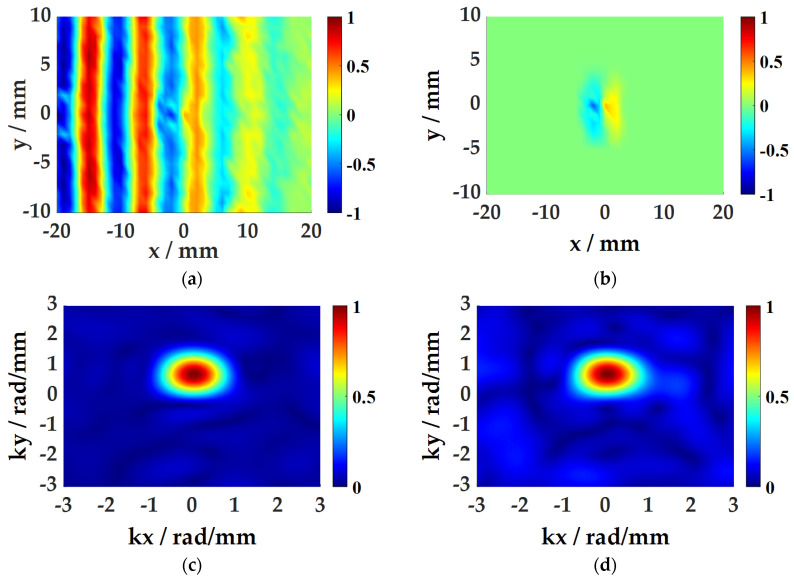
SWA process: (**a**) space-time domain data; (**b**) the windowed space-time domain data; (**c**) wavenumber domain spectrum at 200 kHz; (**d**) wavenumber domain spectrum at 220 kHz.

The spatial window function is used to cut the time–space domain wavefield. Figure 11a,b are the overall wavefield at *t* = 136 μs and the windowed wavefield with the (0,0) coordinate point as the center. Subsequently, the data are subjected to a three-dimensional Fourier transform to obtain wavenumber distribution diagrams at various frequencies. Figure 11c,d are wavenumber distribution diagrams at 200 kHz and 220 kHz. The maximum value of the wavenumber at each frequency is averaged as the actual wave value of the current coordinate point, thereby the wave number diagram of each position in the space is obtained, which can be used to characterize the damage.

### 4.3. Topography Reconstruction Result

#### 4.3.1. Aluminum Plate Damage Imaging

**Figure 12:**
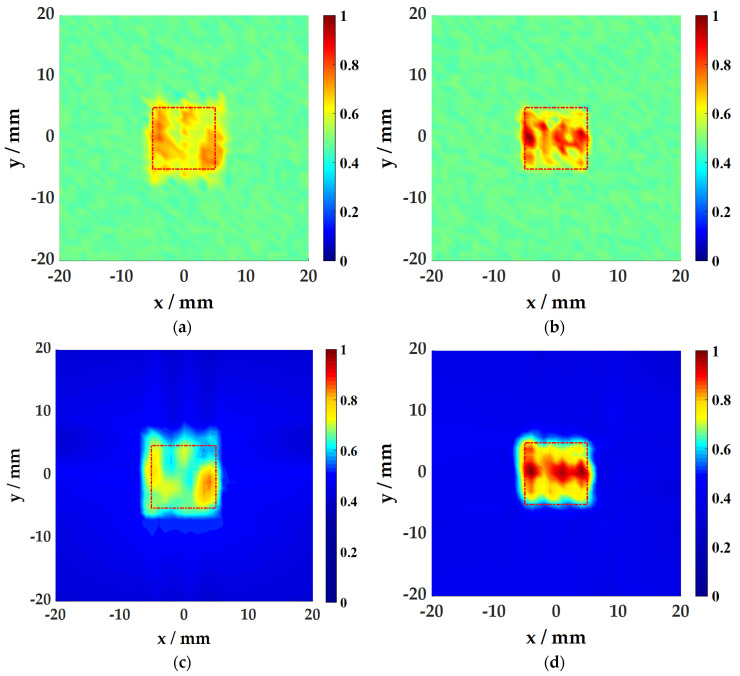
Damage Imaging results of the aluminum plate: (**a**) Imaging of damage at 1 mm depth using the WFA; (**b**) Imaging of damage at 1.5 mm depth using the WFA; (**c**) Imaging of damage at 1 mm depth using the SWA; (**d**) Imaging of damage at 1.5 mm depth using the SWA.

Based on the above two imaging algorithms, the damage of the aluminum plate is imaged, as shown in Figure 12. The topography of the damage is well presented, and the size is consistent with the actual preset size of 10 mm × 10 mm, and the detection size of 1 mm deep damage is larger than 1.5 mm deep damage. For damage edge detection, the SWA imaging results have smoother edges. Comparing the results of damage imaging at two different depths, the energy value and wave value of the damage at 1.5 mm depth are both higher than 1 mm, and the imaging effect is better. This is due to the thinning of the plate thickness in the damaged area, forming an acoustic black hole structure, which causes energy to concentrate, the wave propagation speed becomes slower, and the wave number becomes larger.

#### 4.3.2. CFRP Plate Damage Imaging

**Figure 13:**
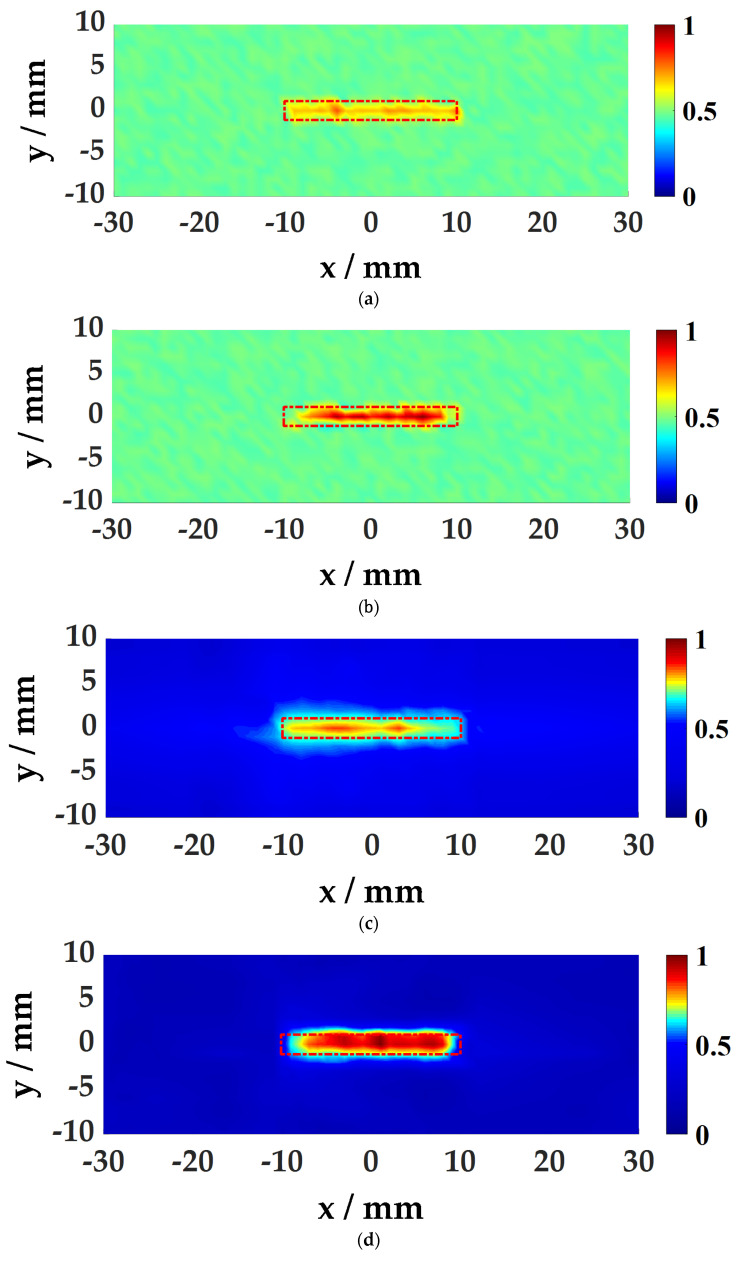
Damage Imaging results of the CERP plate: (**a**) Imaging of damage at 1 mm depth using the WFA; (**b**) Imaging of damage at 1.5 mm depth using the WFA; (**c**) Imaging of damage at 1 mm depth using the SWA; (**d**) Imaging of damage at 1.5 mm depth using the SWA.

Similarly, the damage of the CFRP plate is imaged, as shown in Figure 13. The above detection scheme is still effective for composite materials and elongated damage, and the effect is similar to that of the aluminum plates. Damage topography reconstruction algorithms based on WFA and SWA are applied to the detection of different damage conditions of different plates. Experimental studies have proved that both methods can better present the damage topography and are suitable for damage topography reconstruction of the air-coupled ultrasonic and laser vibrometer hybrid noncontact detection system proposed in this paper. The specific imaging quality evaluation will be carried out in the next section.

### 4.4. Quality Assessment of Damage Imaging Results

In order to specifically evaluate the damage imaging quality, two evaluation indicators, imaging error and image contrast, are used to evaluate the damage imaging effect.

For the two square damages in the aluminum plate, the imaging error *δ*_1_ is represented by the average of the length measurement errors in the *x* and *y* directions. For the damage of two rectangular cracks in the CFRP plate, since the real length in the *y* direction is too small, only the length measurement error in the *x* direction is chosen to represent the imaging error δ_2_.

(11) $$\delta_{1}=\frac{\left(\frac{\left|M_{x}-L_{x}\right|}{L_{x}}+\frac{\left|M_{y}-L_{y}\right|}{L_{y}}\right)}{2},$$

(12) $$\delta_{2}=\frac{\left|M_{x}-L_{x}\right|}{L_{x}},$$

In the above formula:

(13) $$M_{x}=\frac{1}{N}\sum_{i=1}^{N}\left|ML_{i}-MR_{i}\right|,$$

(14) $$M_{y}=\frac{1}{K}\sum_{j=1}^{K}\left|MU_{j}-MD_{j}\right|,$$

Among them, *L_x_* and *L_y_* represent the true value of the length in the *x* and *y* directions respectively, and *M_x_* and *M_y_* represent the measured value of the length in the *x* and *y* directions respectively. *N* and *K* are the number of samples for length measurement in the *x* and *y* directions respectively. *ML*_i_ and *MR*_i_ represent the starting point and end point of measuring the length in the *x* direction in each sample, and *MU*_j_ and *MD*_j_ represent the starting point and end point of measuring the length in the *y* direction in each sample.

In addition to the imaging error defined above, in order to more objectively evaluate the imaging quality of the two algorithms, the Weber contrast is used to evaluate the imaging quality, which is specifically defined as:

(15) $$C=\frac{I-I_{b}}{I_{b}},$$

Among them, *I* and *I_b_* respectively represent the average brightness value of the target in the image and the average brightness value of the background. For Weber contrast, the larger the value, the better the visual contrast between the target and the background, that is, the better the imaging.

Table 2 compares the imaging effects of aluminum plates. First, comparing the P1 and P2 damage detection results under the same algorithm, it can be seen that in WFA, the P2 damage imaging error is reduced by 12.5% from P1, and the contrast is increased by 0.08. In SWA, the P2 damage imaging error is reduced by 9.5%, and the contrast increased by 0.44. The P2 damage is more serious, the more damage information is carried by the collected signal, the more accurate the judgment of the damage, the more accurate the length detection in the *x* and *y* directions of the damage imaging, and the higher the image contrast. Second, comparing the imaging results of different detection algorithms for the same damage, the SWA is slightly better than the WFA. The damage imaging error in the aluminum plate is reduced by 4.5% and 1.5%, and the contrast is increased by 0.13 and 0.49, respectively.

Similarly, the detection effect in the CFRP plate was evaluated, as shown in Table 3, and similar results were obtained. The detection effect of P4 damage is better than that of P3. In the WFA, the imaging error is reduced by 1.5%, and the contrast is increased by 0.49. In the SWA, the imaging error is reduced by 4.0%, and the contrast is increased by 1.72. The damage imaging error of the SWA is reduced by 1% and 3.5% compared with the WFA, and the image contrast of the SWA is better, increasing by 0.91 and 2.14 respectively.

In summary, for the noncontact ultrasonic detection system with air-coupled excitation and laser vibrometer reception proposed in this paper, the SWA is better than the WFA, especially the damage edge detection is smoother and the image contrast is higher. The main reasons are as follows:

(1) WFA uses filtered time-domain sound field data for imaging, but the time-domain data have energy loss, which affects the imaging effect. The SWA is not affected by energy attenuation characteristics. It reflects the damage through the change of wavenumber information and has nothing to do with energy.
(2) The filter function in the WFA has a great influence on the imaging effect, and the selection of the threshold is the core element that determines the imaging quality. When sound waves encounter damage, they will undergo changes such as refraction and reflection, which virtually increases the difficulty of threshold selection. The SWA has stronger stability, does not need to filter the spatial wavenumber, and reduces the cumbersomeness of selecting the filter threshold.
(3) SWA selects a specific frequency range according to the bandwidth of the excitation signal, and averages the signals in the entire frequency band to generate a spatial wavenumber diagram, which basically covers most of the effective data signals, making the imaging results more accurate.

However, although the SWA improves the imaging effect, it also sacrifices a certain detection efficiency. The use of short-space-time three-dimensional Fourier transform makes its calculation time long. In contrast, the WFA does not consider the frequency domain information, and only needs to perform the conversion between the spatial domain and the wavenumber domain. The algorithm calculation time is shorter and the efficiency is higher.

Based on the above test results, air-coupled ultrasound and full-field laser vibrometer detection system has the characteristics of fast scanning, high spatial resolution and complete wave field. It is a structural damage detection system with good development prospects and can be applied to a variety of different algorithms.

## 5. Conclusions

The air-coupled ultrasound and full-field laser vibrometer detection system is proposed in this paper. The air-coupled oblique incident ultrasonic excitation method realizes the generation of single-mode ultrasonic Lamb waves in the aluminum plate and composite plate. The laser vibrometer is used to measure the surface displacement to achieve rapid signal acquisition with high spatial resolution. The above system enables the wavenumber domain analysis methods to be realized in a completely noncontact manner, giving them a broader application scenario. In this paper, aiming at the damaged structure in aluminum and composite thin plates, the damaged topography reconstruction in the plate structure is realized.

Based on the noncontact system, this paper uses multidimensional Fourier transform to analyze the spatial energy, frequency, and wavenumber of the wavefield data in the time–space domain. Through the Wavenumber Filtering Algorithm and Spatial Wavenumber Algorithm, the visual imaging of the damaged area is realized, and completely present the specific size and shape of the damage. Through two evaluation indicators of imaging error and image contrast, the imaging results of different damages on different plates are evaluated, and it is found that the overall detection effect of the SWA is better than that of the WFA. The imaging error reduction amplitude is between 1.0% and 4.5%, and the image contrast of the imaging effect has been effectively improved between 0.1 and 2.2, and the damaged edge of the former is smoother. The advantages and disadvantages of the two imaging algorithms are analyzed, the WFA is greatly affected by the attenuation of signal energy propagation and the threshold selection is complicated. The SWA intuitively reflects the damage situation through the change of the wavenumber, the detection is more stable and the effect is better. However, the SWA process time is long, and the detection efficiency is relatively lower.

Through the above verification and analysis, the noncontact damage detection system based on air-coupled ultrasound and full-field laser vibrometer combines the advantages of two detection methods and is a completely nondestructive structural damage detection system with good development prospects.

## Figures and Tables

**Table 1:** CFRP material parameters.

Density kg/m^3^	Elastic Modulus GPa			Shear Modulus GPa			Poisson’s Ratio		
1600	E_1_	E_2_	E_3_	G_12_	G_13_	G_23_	v_12_	v_13_	v_23_
	172	11.6	11.6	7.8	7.8	3.9	0.36	0.36	0.55

**Table 2:** Evaluation of damage imaging results of aluminum plates.

Damage-Depth	x True Length	y True Length	Method	x Measured Length	y Measured Length	Imaging Error	Weber Contrast
P1-1.0 mm	10 mm	10 mm	WFA	12.6 mm	11.7 mm	21.5%	0.36
			SWA	12.1 mm	11.3 mm	17.0%	0.49
P2-1.5 mm			WFA	11.3 mm	9.5 mm	9.0%	0.44
			SWA	11.2 mm	10.3 mm	7.5%	0.93

**Table 3:** Evaluation of damage imaging results of CFRP plates.

Damage-Depth	x True Length	y True Length	Method	x Measured Length	Imaging Error	Weber Contrast
P3-1.0 mm	20 mm	2 mm	WFA	21.8 mm	9.0%	0.47
			SWA	21.6 mm	8.0%	1.38
P4-1.5 mm			WFA	18.5 mm	7.5%	0.96
			SWA	19.2 mm	4.0%	3.10
